# PGRF: Physics-Guided Rectified Flow for Low-Light RAW Image Enhancement

**DOI:** 10.3390/jimaging11110393

**Published:** 2025-11-06

**Authors:** Juntai Zeng, Qingyun Yang

**Affiliations:** Academy for Advanced Interdisciplinary Studies, Northeast Normal University, Changchun 130024, China; jtceng@nenu.edu.cn

**Keywords:** low-light denoising, noise modeling, rectified flow

## Abstract

Enhancing RAW images acquired under low-light conditions remains a fundamental yet challenging problem in computational photography and image signal processing. Recent deep learning-based approaches have shifted from real paired datasets toward synthetic data generation, where sensor noise is typically simulated through physical modeling. However, most existing methods primarily account for additive noise, neglect multiplicative noise components, and rely on global calibration procedures that fail to capture pixel-level manufacturing variability. Consequently, these methods struggle to faithfully reproduce the complex statistics of real sensor noise. To overcome these limitations, this paper introduces a physically grounded composite noise model that jointly incorporates additive and multiplicative noise components. We further propose a per-pixel noise simulation and calibration strategy, which estimates and synthesizes noise individually for each pixel. This physics-based calibration not only circumvents the constraints of global noise modeling but also captures spatial noise variations arising from microscopic CMOS sensor fabrication differences. Inspired by the recent success of rectified-flow methods in image generation, we integrate our physics-based noise synthesis into a rectified-flow generative framework and present PGRF (Physics-Guided Rectified Flow): a physics-guided rectified-flow framework for low-light RAW image enhancement. PGRF leverages the expressive capacity of rectified flows to model complex data distributions, while physical guidance constrains the generation process toward the desired clean image manifold. To evaluate our method, we constructed the LLID, a dedicated indoor low-light RAW benchmark captured using the Sony A7S II camera. Extensive experiments demonstrate that the proposed framework achieves substantial improvements over state-of-the-art methods in low-light RAW image enhancement.

## 1. Introduction

In recent years, driven by the increasing demand for high-quality visual content, low-light image enhancement has attracted considerable attention in both academia and industry. Deep learning-based approaches have achieved remarkable success and have gradually become the dominant paradigm for this task [1,2,3,4]. These methods typically learn a mapping from low-light images to their corresponding long-exposure (clean) references, demonstrating strong capabilities in detail restoration and noise suppression. Although most existing techniques operate in the sRGB domain and yield visually plausible results, the sRGB space inherently compresses sensor-native information. Consequently, it cannot fully exploit the statistical richness and potential of the underlying sensor data [5]. In contrast, the RAW domain has garnered increasing interest because it retains higher bit depth and directly preserves the sensor’s original noise distribution, thereby providing a more suitable representation for low-light enhancement [6,7]. Nevertheless, enhancing RAW images captured under extremely low illumination remains highly challenging. A major obstacle lies in the requirement for large-scale paired noisy–clean datasets to effectively train learning-based models. Collecting such datasets is labor-intensive, as it demands precise alignment between noisy and clean images without introducing spatial misregistration. To alleviate this difficulty, recent works have explored synthesizing paired datasets by physically modeling the noise formation process of camera sensors and subsequently employing these synthetic pairs for network training, which has proven to be an efficient and practical alternative [8,9,10].

Under low-light conditions, physics-based sensor noise modeling aims to characterize noise by explicitly accounting for the underlying physical generation mechanisms and the statistical properties of the imaging sensor. Existing physics-based approaches, such as ELD [8], typically focus on additive noise while neglecting multiplicative components—namely, the interaction between dark current shot noise and fixed pattern noise. This simplification overlooks critical aspects of real sensor noise formation. Moreover, most prior works rely on frame-level calibration, where a single noise value is used to generate a global noise pattern for the entire sensor (hereafter referred to as global calibration). Such a strategy ignores the fact that, as pixel sizes in modern CMOS sensors shrink, ensuring pixel uniformity becomes increasingly difficult [11]. Consequently, the noise characteristics of individual pixels may differ significantly, leading to inaccuracies in noise modeling when only global calibration is applied. To address these limitations, we propose a composite noise model that integrates both additive and multiplicative components. In addition, we introduce a per-pixel calibration strategy, which replaces global calibration by directly modeling noise behavior at the pixel level. By observing the stochastic characteristics of each pixel and fitting our composite model accordingly, we estimate and synthesize pixel-specific noise patterns. This physics-driven per-pixel noise simulation more faithfully reproduces real sensor noise distributions and, in turn, provides higher-quality paired training samples for learning-based enhancement methods.

Meanwhile, generative models—particularly diffusion-based approaches [12,13]—have demonstrated remarkable advantages in image enhancement by leveraging their strong generative capacity to synthesize fine-grained details. However, their inherently iterative denoising process leads to slow inference, which significantly restricts their deployment in real-time or industrial applications. More recently, the rectified flow generative model (Rectified Flow) [14] has emerged as a promising alternative, offering competitive image quality while enabling fast and efficient generation. Nevertheless, conventional rectified flow models inherently produce a one-to-many mapping, making them ill-suited for low-light image enhancement, which fundamentally requires a one-to-one correspondence between degraded inputs and their enhanced outputs. To overcome this limitation, we propose PGRF (Physics-Guided Rectified Flow), a novel physics-guided rectified flow framework tailored for low-light image enhancement. To mitigate the randomness of rectified flow during generation, and inspired by [12], we design a physics-guided conditional control module that constrains the model, which aims to generate a specific target image consistent with physical priors. Furthermore, based on an in-depth analysis of the rectification strategy in Rectified Flow, we introduce a sampling search strategy that strengthens the model’s ability to accurately model the generation trajectory, thereby enhancing fidelity and stability. By seamlessly integrating physical priors with conditional control and refined sampling, our framework effectively adapts rectified flow to one-to-one mapping tasks and achieves substantial improvements in generation quality for low-light RAW image enhancement.

PGRF eliminates the need for iterative refinement in diffusion models, thereby avoiding long inference times. In addition, the rectified flow formulation effectively estimates the mapping trajectory from a normal distribution to the target image, achieving better image processing results. To the best of our knowledge, this is the first work to integrate physics-guided paired data generation with a rectified flow framework. This unique combination not only alleviates the difficulty of acquiring large-scale paired datasets but also leverages the strong generative capability of rectified flows to achieve superior restoration performance in low-light RAW image enhancement.

Main contributions:

- Pixel-level sensor noise model with multiplicative component.

We develop a pixel-level noise model that extends conventional formulations by explicitly incorporating multiplicative noise. This model provides a more faithful representation of the physical noise generation mechanisms in image sensors and offers a practical pathway for parameter estimation and model optimization.

- Per-pixel calibration and novel noise-simulation strategy.

To accommodate the proposed physical noise model, we move beyond traditional global calibration and introduce a per-pixel calibration scheme. Coupled with a novel noise-synthesis strategy, this approach estimates noise on a pixel-by-pixel basis and generates spatially varying noise patterns. By combining pixel-level calibration with physical modeling, our method achieves more realistic noise reproduction and significantly improves simulation accuracy.

- Physics-Guided Rectified Flow Framework (PGRF).

We integrate physics-based noise modeling with the rectified flow generative paradigm and propose PGRF, a physics-guided rectified flow framework for low-light RAW image enhancement. By leveraging the strong generative capability of rectified flows while constraining them with physical priors, PGRF produces enhanced images that are visually natural and physically consistent.

- A new indoor low-light dataset (LLID).

We construct LLID, an indoor low-light RAW image dataset captured under diverse shooting conditions using the Sony A7S II camera. LLID serves as a benchmark for evaluating enhancement performance and enables comprehensive comparisons across different methods, providing a solid data foundation for future research.

## 2. Recent Work

### 2.1. RAW Low-Light Image Processing

RAW-domain low-light image enhancement has received significant attention in recent years [3,5,7,15]. Chen et al. [3] brought wide recognition to this research area by introducing a dedicated U-Net architecture and establishing the SID dataset, which has since served as a cornerstone for subsequent studies. To address the difficulty of collecting paired RAW data, synthesizing paired datasets has gradually become a mainstream research direction [8,16,17,18,19,20,21,22,23]. In early work, Foi et al. [24] introduced the Poisson–Gaussian noise model for RAW data, modeling image noise as a mixture of Poisson and Gaussian components. While effective in general cases, this model encounters severe challenges under extremely low-illumination conditions [25]. More recently, Wei et al. [8] analyzed the physical imaging mechanisms of camera noise in low-light environments and proposed a physics-based noise model for paired data synthesis. Their method not only enabled the generation of realistic noise distributions but also achieved performance surpassing that of models trained on real paired datasets, thereby drawing considerable attention to physics-based synthetic data approaches.

However, noise modeling approaches based purely on physical principles have predominantly focused on additive noise, thereby neglecting the nonlinear interaction between dark current shot noise and fixed pattern noise—an effect that gives rise to multiplicative noise. The primary reason for this omission lies in the limitations of existing calibration techniques, as accurately estimating multiplicative noise remains highly challenging in practice. To overcome this shortcoming, we adopt a physics-driven perspective and formulate a composite noise model that explicitly incorporates both additive and multiplicative components. Furthermore, we introduce a novel calibration strategy that enables practical estimation of the multiplicative noise term, thereby enhancing the fidelity of the overall noise modeling process.

Abdelhamed et al. [16] introduced Noise Flow, a noise-generation framework based on conditional normalizing flows, which for the first time combined a physics-driven parametric noise model with a deep generative model. Their approach constructs an invertible flow network composed of a signal-dependent layer (to model heteroscedastic noise) and a gain layer (to capture ISO-dependent responses), thereby enabling joint modeling of multi-device and multi-ISO noise distributions on the SIDD dataset. Monakhova et al. [17] proposed a noise modeling strategy that integrates Generative Adversarial Networks (GANs) with physical priors. In this framework, the GAN dynamically adjusts noise parameters according to the sensor-specific noise type, while a discriminator constrains the generated noise to ensure consistency with real sensor characteristics. Similarly, Zhang et al. [19] combined physical modeling with GANs and proposed a general noise model with a decoupled synthesis process. Their method employs a generative network for noise synthesis and utilizes a Fourier-transform-based discriminator to evaluate whether the synthesized noise conforms to sensor noise statistics. More recently, Qin et al. [22] adopted diffusion models for noise modeling, where noise is progressively added through multiple steps and subsequently reversed via iterative denoising. Li et al. [26] presented a hypothesis-driven shot noise synthesis method that generates realistic noise patterns using only captured dark frames, thereby eliminating the need for extensive paired datasets.

Among existing approaches, learning-based methods typically train neural networks on real paired datasets to construct surrogate models of sensor noise. However, their modeling performance is constrained by the inaccuracy of distribution estimation mechanisms [23]. In addition, the heavy reliance on large-scale paired datasets directly conflicts with our objective of avoiding the costly and labor-intensive process of dataset collection. For this reason, our work primarily emphasizes physics-based sensor noise modeling, which circumvents the need for paired training data by estimating sensor noise directly from physical principles. This perspective not only reduces the dependence on dataset acquisition but also provides a more principled and interpretable foundation for modeling noise in low-light RAW image enhancement.

### 2.2. Low-Light Image Enhancement Based on Generative Models

In recent years, Generative Adversarial Network (GAN)-based methods have received considerable attention for low-light image enhancement [27,28,29]. Despite their success, GAN-based approaches often suffer from inherent instability, sensitivity to hyperparameters, and difficulties in training convergence, which pose substantial challenges in practice. With the advent of diffusion models [30], their strong generative capacity and enhanced training stability have gradually established them as a mainstream framework for image generation. Leveraging their ability to synthesize high-quality image details, recent studies have increasingly explored the application of diffusion models to low-light image enhancement [5,12,13,31], demonstrating promising results compared to earlier GAN-based methods.

Zhou et al. [12] addressed the challenges of complex sampling procedures and long inference times in diffusion models by proposing a pyramid diffusion model, which achieved notable improvements in both sampling efficiency and restoration quality. Hou et al. [13] introduced a curvature regularization term into the diffusion process to constrain the curvature of the corresponding ODE trajectory. By maintaining a low-curvature path, their approach prevented excessive divergence and instability during generation. In addition, they incorporated non-local structural information into a global structure-aware regularization term, which preserved overall structural consistency while enhancing image contrast and detail representation. Jiang et al. [31] combined diffusion models with wavelet transforms and proposed a wavelet-based conditional diffusion model. In this framework, the input image was decomposed using multi-level two-dimensional discrete wavelet transforms, restricting the diffusion process to the wavelet domain. This design effectively compressed spatial resolution, reduced computational complexity, and significantly accelerated inference speed. Li et al. [5] explored diffusion models for RAW-domain low-light image enhancement and proposed a two-stage framework consisting of pre-training and alignment. In the pre-training stage, multiple virtual camera noise models were constructed in the noise space, and a Camera Feature Integration (CFI) module was introduced to learn cross-camera general feature representations. During the alignment stage, a small number of real paired RAW images were used for fine-tuning to adapt the model to device-specific noise characteristics. To further mitigate color shifts commonly observed in diffusion processes, they incorporated a color corrector that dynamically adjusted the global color distribution, thereby improving color consistency in the final enhanced image. Lu et al. [32] proposed NoiseDiff, a diffusion-based low-light noise synthesis method that effectively models the complex distribution of real noise. The synthetic data generated by NoiseDiff significantly improves the performance of low-light image denoising.

## 3. Mathematical Modeling of Image Sensors

Our analysis primarily focuses on CMOS image sensors. We begin by examining the underlying physical mechanisms through which individual sensor pixels generate noise. Building upon this analysis, we construct a mathematical composite noise model that incorporates both additive and multiplicative components, thereby providing a more accurate characterization of noise at the pixel level. The proposed noise model is specifically designed for low-light imaging conditions and assumes that the exposure time during image acquisition exceeds the sensor’s minimum exposure threshold. Under this condition, characteristic noise patterns emerge, which are effectively captured by our formulation.

### 3.1. Noise Analysis

#### 3.1.1. Photon Shot Noise

Photon shot noise, also referred to as quantum noise, originates from the discrete and statistical nature of photons. Since photons exhibit quantum particle behavior, their arrival at the image sensor follows a random process. As a result, the number of incident photons fluctuates around an expected mean value rather than remaining constant, thereby introducing noise into the captured signal. Photon shot noise is widely recognized to follow a Poisson distribution [33]. Mathematically, it can be expressed as:

(1) $$\left(I(x,y)+N_{s}(x,y)\right)∼P\left(\lambda \right)$$

where (x,y) represents the pixel coordinates, I represents the true photon count of the target scene, and $N_{s}$ represents the photon shot noise component. This type of noise is intrinsic to the fundamental properties of light and is therefore unavoidable in image sensing.

#### 3.1.2. Read Noise

Read noise is defined as the collection of noise components that remain independent of the incident light signal [34]. It encompasses multiple sources, including dark current shot noise, fixed-pattern noise (FPN), row noise, source follower noise, and quantization noise. Collectively, these noise sources originate from electronic circuitry and sensor architecture rather than the photon arrival process, and thus they are considered signal-independent contributions to the overall noise in CMOS image sensors.

#### 3.1.3. Dark Current Shot Noise

Dark current primarily arises from thermally generated electron–hole pairs within the semiconductor material. Even in the absence of illumination, these electrons and holes are randomly generated and migrate within the sensor, thereby forming a background current commonly referred to as dark current.

The physical expression of dark current is given in [35] as:

(2) $$S=QT^{3/2}C\exp\left(-\frac{E_{gap}}{2\cdot kT}\right)$$

where Q denotes the pixel area, T represents the absolute temperature, and C is the dark current figure-of-merit at 300 K, which varies across different sensors and is typically specified by the manufacturer. $E_{gap}$ denotes the semiconductor band gap energy, and k is the Boltzmann constant.

This type of noise induced by dark current is referred to as dark current shot noise. It originates from structural defects introduced during the semiconductor fabrication process. Consequently, the magnitude of dark current varies across different pixels and also depends on the exposure time—specifically, under fixed conditions for other parameters, a longer exposure time results in stronger dark current noise. Dark current shot noise is generally modeled as following a Poisson distribution [36]. Its formulation can be expressed as:

(3) $$N_{RS}\left(x,y\right)=t\cdot S$$

In probabilistic form, this noise is modeled as:

(4) $$N_{RS}\left(x,y\right)∼P\left(\lambda \right)$$

where $N_{RS}$ represents the dark current shot noise.

#### 3.1.4. Fixed-Pattern Noise

Fixed-pattern noise (FPN) primarily arises from manufacturing process variations in image sensors, such as non-uniform transistor threshold voltages and doping concentrations, which lead to pixel-dependent deviations. In captured images, FPN manifests as fixed noise points at specific pixel locations. Unlike random noise components, FPN remains stable across consecutive frames and therefore cannot be effectively suppressed by temporal averaging. A common strategy for mitigating FPN is to subtract the bias estimated from dark-frame captures. However, under low-light conditions, FPN becomes more pronounced and exerts a stronger detrimental effect on image quality. From a sensor-wide perspective, FPN is generally modeled as following a Gaussian distribution [25], expressed as:

(5) $$N_{FP}\left(x,y\right)∼N\left(0,\sigma_{FP}^{2}\right)$$

where $N_{FP}$ represents the fixed-pattern noise (FPN).

Since dark current shot noise follows a Poisson distribution, we treat it as a form of multiplicative noise. Inspired by [25], we describe the interaction between dark current shot noise and fixed-pattern noise using the following relation:

(6) $$N_{d}=N_{RS}\left(x,y\right)+N_{RS}\left(x,y\right)\cdot N_{FP}\left(x,y\right)$$

where $N_{d}$ denotes the total contribution of dark current shot noise and fixed-pattern noise.

#### 3.1.5. Row Noise

In CMOS sensors, each row is typically controlled by a dedicated analog-to-digital converter (ADC) during the signal readout process. Slight fluctuations or instabilities in the operating voltages of different ADCs result in inconsistencies in signal intensity across rows, thereby giving rise to row noise. Row noise is generally modeled as following a Gaussian distribution [37], expressed as:

(7) $$N_{H}\left(x,y\right)∼N\left(0,\sigma_{H}^{2}\right)$$

where $N_{H}$ denotes the row noise distribution.

#### 3.1.6. Source Follower Noise

Source follower noise primarily originates from defects associated with the random capture and release of charge carriers in the silicon lattice. It is mainly composed of thermal noise, flicker noise (1/f noise), and random telegraph noise (RTN). Collectively, these fluctuations degrade the sensor’s readout stability. This type of noise is generally modeled as a Gaussian distribution [37], expressed as:

(8) $$N_{r}\left(x,y\right)∼N\left(0,\sigma_{r}^{2}\right)$$

where $N_{r}$ denotes the source follower noise.

#### 3.1.7. Quantization Noise

Quantization noise arises during the analog-to-digital conversion (ADC) process, where continuous analog signals are discretized into a finite number of digital levels. This error manifests as the difference between the original input signal and its quantized representation. Due to the limited precision of the sensor’s ADC, signals are quantized before being stored as RAW RGB images [18]. Quantization noise is generally modeled as following a uniform distribution, expressed as:

(9) $$N_{q}\left(x,y\right)∼U(-1/2q,1/2q,)$$

where $N_{q}$ denotes the quantization noise, q is the quantization step size, which is typically 1.

### 3.2. Model Establishment

#### 3.2.1. Noise Relationship Analysis

Based on the above analysis of various noise sources, we establish the following composite noise model that integrates both additive and multiplicative components:

$$D(x,y)=K\left(I(x,y)+N_{s}(x,y)\right)+N_{RS}(x,y)\left(1+N_{FP}(x,y)\right)+N_{H}(x,y)+N_{r}(x,y)+N_{q}(x,y)$$

Here, (x,y) represents the pixel coordinates; $N_{s}$ represents photon shot noise; $N_{FP}$ represents fixed-pattern noise; $N_{RS}$ represents dark current shot noise; $N_{H}$ represents row noise; $N_{r}$ represents source follower noise; $N_{q}$ represents quantization noise; and K represents the photon-to-electron conversion rate inside the image sensor.

#### 3.2.2. Calibration Method

To solve for the parameters of the proposed noise model and precisely characterize the noise properties of CMOS image sensors, we employ three types of RAW images: flat-field frames, bias frames, and dark frames. Each type of frame is designed to capture distinct noise sources and serves a specific calibration purpose.

- Flat-field frame: This frame is captured under uniform illumination, where the sensor is directed toward a homogeneously lit white surface. To facilitate subsequent computations, the exposure time is set identical to that of the bias frame, and the focal length is adjusted to infinity. The flat-field frame is primarily used to calibrate the uniformity of the sensor response and provides a reference baseline for subsequent noise analysis.
- Bias frame: The bias frame is obtained at the shortest exposure time with the sensor lens fully covered, producing a completely dark image. It captures time-independent noise introduced by the sensor circuitry, including fixed-pattern noise and source follower noise.
- Dark frame: The dark frame is captured under the same exposure time, ISO setting, and temperature conditions as the flat-field frame, but with the lens covered. In addition to the noise components present in the bias frame, it also incorporates dark current shot noise induced by the integration time. Dark frames therefore provide an effective means of characterizing dark current noise under long-exposure conditions and are essential for high-precision noise calibration.

In simulating low-light image sensor noise, global calibration is commonly adopted to guide the noise simulation process. While this strategy reduces computational complexity, it suffers from notable limitations in terms of simulation accuracy. During the manufacturing process, image sensors inevitably exhibit process variations, resulting in subtle differences in the quantum efficiency and charge collection efficiency of individual pixels. These variations lead to pixel-dependent noise characteristics. However, global calibration neglects such pixel-level differences, which ultimately degrades the accuracy of noise reproduction and adversely impacts image quality and fidelity under low-light conditions.

**Figure 1:**
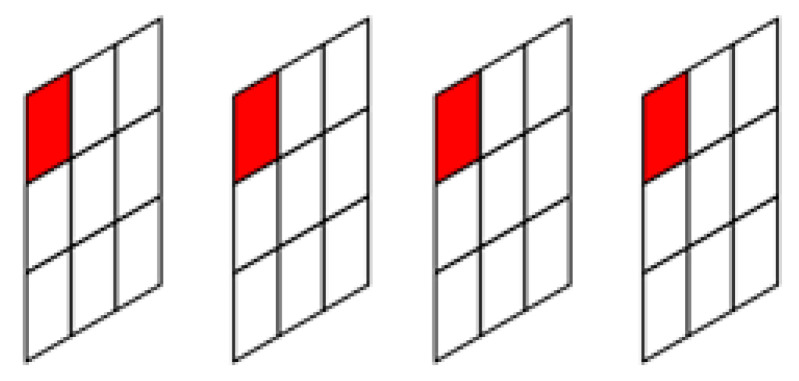
Red dots represent the pixel values at corresponding positions across frames.

To overcome the limitations of global calibration, we propose a novel noise-fitting method that performs fine-grained calibration of noise characteristics at the pixel level and generates noise in a targeted manner. Specifically, the calibration is carried out spatially by computing the mean and variance of each pixel at its corresponding position across multiple frames, thereby capturing pixel-specific noise statistics. This process enables per-pixel modeling of noise behavior, as illustrated in Figure 1.

**Figure 2:**
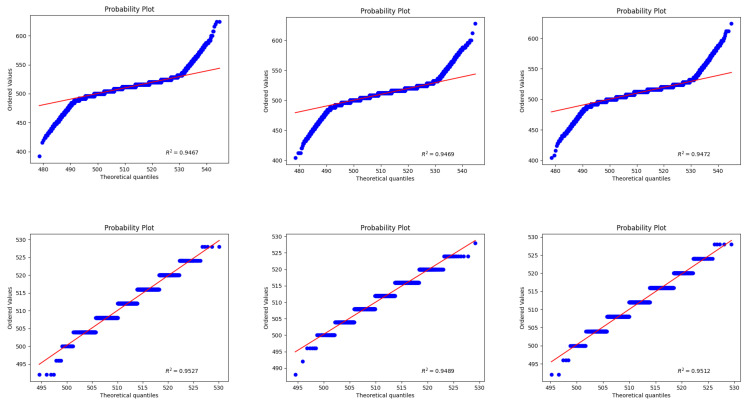
Variance distribution of source follower noise under ISO 800. The upper panel shows the results obtained by global fitting, while the lower panel presents the results obtained by single-pixel fitting. The blue points represent the quantiles of the real noise distribution, and the red line represents the quantiles of the synthetic noise distribution.

To evaluate the effectiveness of the proposed pixel-wise fitting strategy, we compare it against conventional single-frame fitting. Specifically, we randomly select pixel positions and fit their noise distributions using the Probability Plot Correlation Coefficient (PPCC) [38]. The resulting $R^{2}$ value reflects the goodness of fit, with larger values indicating better fitting performance. As shown in Figure 2, in most cases, the pixel-wise noise fitting achieves higher $R^{2}$ values and thus outperforms conventional single-frame fitting, demonstrating its superiority in accurately capturing pixel-level noise characteristics.

**Figure 3:**
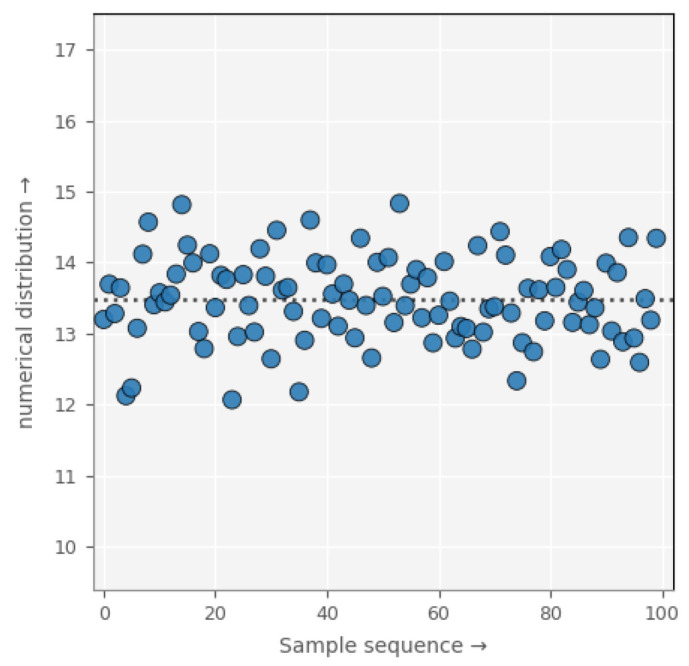
Variance distribution of source follower noise across individual pixels. The blue dots represent the variance of individual pixels, and the dashed line represents the mean of all variances.

This fine-grained, pixel-level calibration method not only significantly improves the accuracy of noise simulation but also underscores its practical importance by revealing substantial variance differences across pixels. As illustrated in Figure 3, we randomly selected 100 pixels and compared their variances, which exhibited an amplitude difference of approximately 3. However, when subjected to post-processing with magnification factors ranging from 100 to 200, mathematical analysis revealed that the variance amplification could reach levels between 10,000 and 40,000 times. These findings further confirm the critical importance of pixel-level calibration in low-light imaging, as even small pixel-level deviations can be dramatically magnified during subsequent image processing, severely degrading image quality if left uncorrected.

Compared with traditional approaches that approximate noise using a single global variance value, the proposed pixel-wise noise generation enables more precise and targeted modeling of sensor noise. This fine-grained strategy provides a more faithful reproduction of real noise characteristics, thereby offering robust technical support for achieving high-quality imaging under low-light conditions.

Since fixed-pattern noise (FPN) remains constant across frames, its value is estimated by averaging each pixel’s intensity over the acquired bias frames. In contrast, source follower noise is estimated by computing the variance across individual pixels within the bias frames. Once the photon conversion coefficient K and the dark current shot noise are estimated through the proposed model, the calibrated data can then be employed to synthesize realistic sensor noise, thereby enabling accurate noise reproduction under low-light conditions.

#### 3.2.3. Model Solution

We divide the solution of the proposed noise model into two parts according to whether the noise components are illumination-dependent. The first part concerns the calibration of photon shot noise, which is primarily estimated using flat-field frames. The second part focuses on the calibration of readout noise, which is derived from dark frames and bias frames.

Formally, the overall formulation can be expressed as:

$$D\left(x,y\right)=K\left(I(x,y)+N_{s}(x,y)\right)+N_{RS}(x,y)\left(1+N_{FP}(x,y)\right)+N_{H}(x,y)+N_{r}(x,y)+N_{q}(x,y)$$

For simplicity of representation, the noise model can be rewritten in the following compact form:

(10) $$D\left(x,y\right)=K\left(I+N_{s}\right)+N$$

(11) $$N=N_{RS}\cdot \left(1+N_{FP}\right)+N_{H}+N_{r}+N_{q}$$

#### 3.2.4. Photon Shot Noise Calibration

We begin by estimating the unknown variables in Equation (10), and subsequently treat Equation (11) as a whole for the estimation of its unknowns. In the first step, our primary objective is to estimate the coefficient K. Following the approach in [8], we start by taking the variance on both sides of Equation (10):

(12) $$Var\left(D\right)=Var\left(K\left(I+N_{s}\right)\right)+Var\left(N\right)$$

Since $\left(I+N_{s}\right)∼P\left(\lambda \right)$, its variance equals its mean. Therefore:

(13) $$Var\left(D\right)=K^{2}I+Var\left(N\right)$$

By simplifying Equation (13), we obtain:

(14) $$\frac{Var\left(D\right)-Var\left(N\right)}{\left|K\right|I}=\left|K\right|$$

Given that $Var\left(D\right)\gg Var\left(N\right)$, Equation (14) can be approximated as:

(15) $$\frac{Var\left(D\right)}{\left|K\right|I}=\left|K\right|$$

Here, $Var\left(D\right)$ is estimated using the variance of the flat-field frame, while $\left|K\right|I$ is estimated using the mean of the flat-field frame. Therefore, all terms on the left-hand side of Equation (15) are known, which allows us to estimate the coefficient K.

#### 3.2.5. Readout Noise Calibration

As discussed in Section 3.1.3 and Section 3.1.4, when performing calibration across the entire image sensor, dark current shot noise follows a Poisson distribution, while fixed-pattern noise follows a Gaussian distribution. Consequently, Equation (11) takes the form of a Poisson distribution multiplied by a Gaussian distribution, which renders direct solution intractable. However, since our calibration is performed at the individual pixel level, we can exploit the property noted in Section 3.1.4—that the fixed-pattern noise of each pixel remains constant. Therefore, it can be treated as a constant term, denoted as $Q=\left(1+N_{FP}\right)$. By taking the mean of both sides of Equation (11), the expression can be simplified as follows:

(16) $$E\left(N\right)=E\left(N_{RS}\cdot \left(1+N_{FP}\right)\right)+E\left(N_{h}\right)+E\left(N_{r}\right)+E\left(N_{q}\right)$$

This can be further simplified as:

(17) $$E\left(N\right)=Q\cdot E\left(N_{RS}\right)$$

Here, $E\left(N\right)$ denotes the mean of the dark frame. Since the mean of a Poisson distribution is always non-negative, we take the absolute value of the estimated $N_{RS}$ to obtain the estimated dark current shot noise.

## 4. Enhanced Model Based on Rectified Flow

Given the remarkable performance of rectified flow generative models in image generation and processing, our objective is to harness their strong generative capability for RAW low-light image enhancement, while constraining the generation process under the guidance of physics-based noise models.

### 4.1. Introduction to Rectified Flow

Rectified Flow [14] is a method that constructs transport mappings between two probability distributions by learning an ordinary differential equation (ODE) trajectory that closely follows a linear interpolation path. This approach is characterized by its simplicity and efficiency, and has been widely applied to tasks such as generative modeling and domain transfer. Specifically, let two probability distributions defined on the Euclidean space $R^{d}$ be denoted as the source distribution $\pi_{0}$ and the target distribution $\pi_{1}$. The transport mapping problem aims to find a deterministic mapping $T:R^{d}\to R^{d}$ such that, for any $X_{0}∼\pi_{0}$, it holds that $T\left(X_{0}\right)∼\pi_{1}$.

Rectified flow realizes the transport mapping from $\pi_{0}$ to $\pi_{1}$ by constructing a continuous-time ODE flow along a straight-line interpolation path. Specifically, let $X_{0}∼\pi_{0}$, $X_{1}∼\pi_{1}$, then the linear interpolation path over time $t\in \left[0,\;1\right]$ is defined as:

(18) $$X_{t}=tX_{1}+\left(1-t\right)X_{0}$$

where $X_{t}$ denotes the intermediate state at time *t*.

This path corresponds to the shortest trajectory in Euclidean space between $X_{0}$ and $X_{1}$. The core idea of Rectified Flow is to fit a parameterized velocity field $v_{\theta }\left(z,t\right)$, which defines the following form of an ordinary differential equation (ODE) in the continuous-time domain:

(19) $$\frac{dX_{t}}{dt}=v_{\theta }\left(X_{t},t\right)$$

Here, $X_{t}$ denotes the trajectory of a random variable evolving with time, and $v_{\theta }$ parameterized by a neural network, estimates the vector field that best approximates the velocity of the interpolation path. Since $X_{t}$ is the linear interpolation between $X_{0}$ and $X_{1}$, its derivative is constant:

(20) $$\frac{dX_{t}}{dt}=X_{1}-X_{0}$$

Therefore, rectified flow fits the velocity field $v_{\theta }$ such that its output along the interpolation path aligns as closely as possible with this derivative direction. Concretely, this differential equation describes the continuous transport process from the source distribution $\pi_{0}$ to the target distribution $\pi_{1}$. By differentiating Equation (18) with respect to t, the target direction $X_{1}-X_{0}$ is obtained, and the velocity field $v_{\theta }$ can then be fitted by solving the following least-squares optimization problem:

(21) $$\underset{\theta }{\min}\int_{0}^{1}E{\left\|\left(X_{1}-X_{0}\right)-v_{\theta }\left(tX_{1}+\left(1-t\right)X_{0},t\right)\right\|}^{2}dt$$

where $v_{\theta }$ denotes the velocity field parameterized by a neural network with parameters θ.

This objective can be efficiently optimized using standard stochastic gradient descent (SGD), without the need for adversarial training, variational inference, or other forms of complex approximate reasoning. As a result, Rectified Flow not only establishes a theoretically stable generative model but also, in practice, significantly reduces the number of inference steps. This leads to notable improvements in both the efficiency and quality of image generation and cross-distribution mapping.

### 4.2. RAW Image Enhancement Based on Rectified Flow

Rectified flow models have attracted considerable attention due to their stability and strong generative capability. However, their inherent stochasticity poses challenges when directly applied to deterministic tasks such as image enhancement. Inspired by [39], we introduce guiding conditions into rectified flow to enable effective recovery of target images. Since the physical noise model in low-light environments inherently encodes sensor-specific noise characteristics, our objective is to train the velocity field under the joint guidance of noisy images generated from this model. In this way, the rectified flow is constrained to recover the corresponding clean target image with higher fidelity. Based on this principle, we construct a Physics-Guided Rectified Flow (PGRF) framework, which integrates physics-based modeling with rectified flow generative learning for low-light RAW image enhancement.

First, we define the base model as:

(22) $$X_{t}=tX_{H}+\left(1-t\right)X_{0}$$

(23) $$v_{\theta }=f\left(X_{t},t\right)$$

Here, $X_{H}$ denotes a high-quality image, while $X_{0}$ represents a noise sample drawn from a standard normal distribution, and $t\in \left[0,\;1\right]$, f serves as the transformation module that maps between the latent noise space and the image domain.

To enhance the network’s capability for low-light image enhancement, we introduce guiding conditions into the model. Specifically, based on the noise distribution characteristics of low-light images, we design a noise modeling module that injects physically inspired noise into high-quality images. This process simulates noisy images $X_{L}$ produced under low-light conditions, which can be expressed as:

(24) $$X_{L}=C\left(X_{H}\right)$$

where $C\left(\cdot \right)$ denotes the noise addition module, $X_{H}$ is the clean high-quality image, and $X_{L}$ is the corresponding synthetic low-light noisy image.

Accordingly, we obtain the following guiding condition:

(25) $$Τ=(X_{L})$$

We then integrate the guiding condition into the processing network f, yielding

(26) $$v_{\theta }=f\left(X_{t},Τ,t\right)$$

Subsequently, the trajectory predicted by the velocity field $v_{\theta }$ is optimized by minimizing the following loss function:

(27) $$L=\min{\left\|X_{H}-X_{0}-v_{\theta }\left(X_{t},Τ,t\right)\right\|}_{1}$$

### 4.3. Sampling Search Strategy

Inspired by [40], which observed that rectified flow models exhibit varying levels of training difficulty across different sampling timesteps, we further hypothesize that prediction quality during the sampling phase may also vary significantly across timesteps. In other words, certain timesteps along the sampling trajectory may play a more critical role in determining the final generation quality. Based on this hypothesis, we propose a sampling step search strategy that aims to improve inference performance without modifying the pretrained model parameters. This strategy adaptively identifies and emphasizes the most informative timesteps during sampling, thereby enhancing both the efficiency and quality of low-light image enhancement.

Specifically, after training is completed, we freeze the model parameters and construct the guiding conditions using high-quality images (the construction method of these guiding conditions is detailed in Section 4.2). During sampling, instead of adopting the random timestep sampling strategy used in training, we introduce a structured equidistant sampling strategy. By controlling the sampling step size s and the total number of steps n, the sampling timestep sequence is generated as:

$$t=\left\{s,2s,3s\ldots ,ns\right\},\;0<s<1,\;0<ns<1$$

where s and n are tunable parameters that determine the granularity and length of the sampling process.

After performing step-size search and obtaining the optimal sampling time $t_{2}$, we employ a two-stage sampling mechanism during testing to fully exploit the performance gains from the search. First, we set the time step to $t_{1}=0$, and then obtain the intermediate image $X_{Z}$ based on Equation (18) and the trained velocity field $v_{\theta }$.

(28) $$X_{Z}=v_{\theta }\left(X_{0},Τ,t_{1}\right)+\left(1-t_{1}\right)X_{0}$$

Next, the intermediate result $X_{Z}$ is combined with the original input $X_{0}$ through the sampling timestep $t_{2}$ to construct an intermediate state $X_{t}$:

(29) $$X_{t}=t_{2}X_{Z}+\left(1-t_{2}\right)X_{0}$$

Finally, using the optimal timestep $t_{2}$ obtained from the search, we invoke the rectified flow prediction process again to generate the final output image $X_{M}$:

(30) $$X_{M}=v_{\theta }\left(X_{t},Τ,t_{2}\right)+X_{0}$$

The essence of this sampling scheme lies in first obtaining a stable intermediate state at timestep $t_{1}=0$, and then leveraging the optimal timestep $t_{2}$ for a second-stage refinement. This hierarchical two-stage sampling not only preserves the strong generative capability of the rectified flow model but also significantly enhances controllability along the sampling trajectory, thereby improves fine detail reconstruction in the generated images. The specific pseudocode is presented in Algorithms 1–3.
**Algorithm 1** Training1:$\mathrm{Input}:\;\mathrm{target}\;\mathrm{image}\;X_{1}$$;\;\mathrm{velocity}\;\mathrm{model}\;v_{\theta }$$:\;R^{d}\to R^{d}$ withparameterθ;  Physics-Based Noise Model C(X)2:$\mathrm{Sample}:\;X_{0}∼N\left(0,1\right)$3:$\mathrm{Sample}:\;T=C\left(X_{1}\right)$4:$\mathrm{Sample}:\;t∼Uniform(\left[0,1\right])$5:$X_{t}=tX_{1}+\left(1-t\right)X_{0}$6:$\overset{\wedge }{\theta }=\min{\left\|X_{1}-X_{0}-v_{\theta }\left(X_{t},Τ,t\right)\right\|}_{1}$7:$dZ_{t}=v_{\overset{\wedge }{\theta }}\left(Z_{t},T,t,\;\right)dt$8:$\mathrm{Return}:\;Z=\left\{Z_{t}:t\in \left[0,\;1\right]\right\}$
**Algorithm 2** Sampling1:$\mathrm{Input}:\;\mathrm{target}\;\mathrm{image}\;X_{1}$$;\;\mathrm{velocity}\;\mathrm{model}\;v_{\theta }$$:\;R^{d}\to R^{d}$ with parameter θ; Physics-Based Noise Model C(X)$;\;t_{best}=0$$;\;M_{best}=0$.2:Repeat3:$\mathrm{Sample}:\;X_{0}∼N\left(0,1\right)$4$\mathrm{Sample}:\;T=C\left(X_{1}\right)$5:$X_{2}=v_{\theta }\left(X_{0},Τ,0\right)+X_{0}$6:for t=s,2s,3s…,ns do; 0<s<1, 0<ns<17:$X_{t}=tX_{2}+\left(1-t\right)X_{0}$8:$X_{M}=v_{\theta }\left(X_{t},Τ,t\right)+X_{0}$9:M$=P\left(X_{M},X_{1}\right)$ $P\left(X_{M},X_{1}\right)$$\;\mathrm{means}\;\mathrm{computing}\;\mathrm{the}\;\mathrm{PSNR}\;\mathrm{between}\;X_{M}$$\;\mathrm{and}\;X_{1}$10:If M$\;>\;M_{best}$;11:$M_{best}\;=\;$M12:$t_{best}\;=\;$t13:$\mathrm{Return}\;t_{best}$


**Algorithm 3** Testing1:$\mathrm{Input}:\;\mathrm{Low-light}\;\mathrm{image}\;X_{3}$$;\;\mathrm{optimal}\;\mathrm{sampling}\;\mathrm{step}\;\mathrm{size}\;t_{best}$$;\;\mathrm{velocity}\;\mathrm{model}\;v_{\theta }$$:\;R^{d}\to R^{d}$ with parameter θ; R denotes the magnification factor.2:

$\mathrm{Sample}:\;X_{0}∼N\left(0,1\right)$

3:

$\mathrm{Sample}:\;T=X_{3}$

4:

$X_{2}=v_{\theta }\left(X_{0},Τ,0\right)+X_{0}$

5:

$X_{t}=t_{best}X_{2}+\left(1-t_{best}\right)X_{0}$

6:

$X_{M}=v_{\theta }\left(X_{t},Τ,t_{best}\right)+X_{0}$

7:

$\mathrm{Return}\;X_{M}$




## 5. Experiments

### 5.1. Dataset Construction

For our indoor low-light dataset LLID, we provide a paired noisy–clean dataset captured using a Sony A7S II camera (Sony Corporation, Tokyo, Japan). To eliminate errors caused by camera shake and focus instability, the camera was remotely controlled via Sony’s official software Imaging Edge Desktop software (version 1.2.01.04031, Sony Corporation, Tokyo, Japan), ensuring no physical contact occurred during the image acquisition process.

For image acquisition, we followed a strategy similar to that used in ELD [8]: noisy images were captured at high ISO values with short exposure times, while the corresponding clean reference images were obtained at the same ISO levels but with long exposure times. The dataset covers 10 indoor scenes and includes three ISO levels (800, 1600, and 3200) for testing. In total, it consists of 160 paired images. This dataset provides a reliable benchmark for evaluating low-light RAW image enhancement methods, as it captures realistic sensor noise characteristics under varying ISO conditions.

### 5.2. Verification of the Physical Model

In this section, we provide a detailed description of the experimental setup and present comprehensive evaluations. We compare our method against existing physics-based approaches to demonstrate the superiority of the proposed model and methodology.

#### 5.2.1. Experimental Details

We adopted the same noise simulation strategy as ELD [8], where low-light images are simulated by downscaling clean images by a factor of 200–300. Subsequently, noise is randomly generated and added to the downscaled images based on the variances of the different noise components estimated using our model. This procedure ensures that the simulated noisy images faithfully reproduce the statistical properties of real sensor noise under low-light conditions.

For pixel-level noise calibration, we utilize the central 1024 × 1024 region of the captured flat-field, dark-field, and bias images. For row noise calibration, which requires computing the standard deviation of the mean pixel values across sensor rows, we adopt a global calibration approach when generating row noise. In contrast, fixed-pattern noise, dark current shot noise, source follower noise, and photon shot noise are all generated on a per-pixel basis to accurately capture pixel-wise noise variations.

When estimating dark current shot noise, to avoid frequently capturing dark frames with varying exposure times, we chose to capture dark frames with relatively long exposure times to estimate the dark current shot noise. Based on the noise electron generation mechanism within the image sensor [25], demonstrates that dark current shot noise is proportional to time t. According to Equation (2), it can be fitted using the linear relationship y=at. However, since the noise generated by the image sensor undergoes complex internal conversions—from electrons to voltage and numerical quantization during readout—the corresponding relationship may vary. Therefore, after comprehensive analysis, we propose using the nonlinear formula $y=a\sqrt{t}$ to better fit the shot noise. The comparative results, as presented in Table 1, validate the effectiveness of our proposed fitting approach. Furthermore, to improve robustness across different exposure times under the same ISO setting, we employ random sampling of exposure times during simulation to ensure a more accurate and generalized noise estimation.

**Figure 4:**
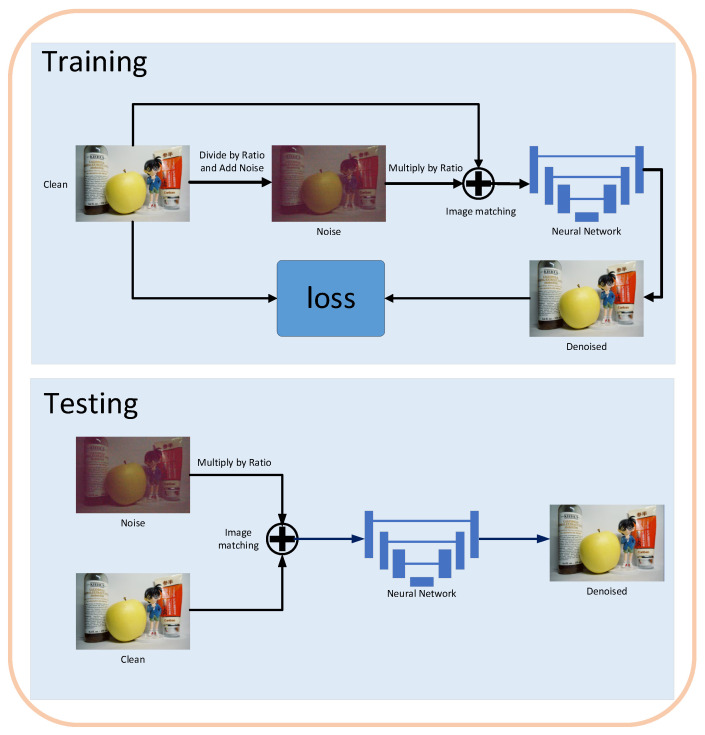
Overview of the image training and testing pipeline used in our experiments.

This study focuses on calibrating individual pixel points of image sensors. Due to the differences in pixel-level noise characteristics across different cameras, the network trained using our method may exhibit limited generalization ability. To validate the accuracy of the proposed noise model for low-light noise modeling, we conducted experiments on the LLID. For fair comparison, we adopted the same deep neural network architecture as in [8] for low-light image denoising. Specifically, [8] employed a U-Net architecture identical to that in [3,41], which has been widely used in image denoising tasks. We selected long-exposure images from the SID dataset as the training set, assuming these long-exposure images to be ideal clean references. Based on this assumption, we synthesized corresponding noisy counterparts using the established noise model, thereby generating noise-injected samples for training. Our implementation is based on PyTorch 3.6. We employed an $L_{1}$ loss function and the Adam optimizer with a batch size of 1, training the model for 200 epochs. The initial learning rate was set to $1.0\times 10^{-4}$. The overall training and testing pipeline is illustrated in Figure 4.

#### 5.2.2. Experimental Results

**Figure 5:**
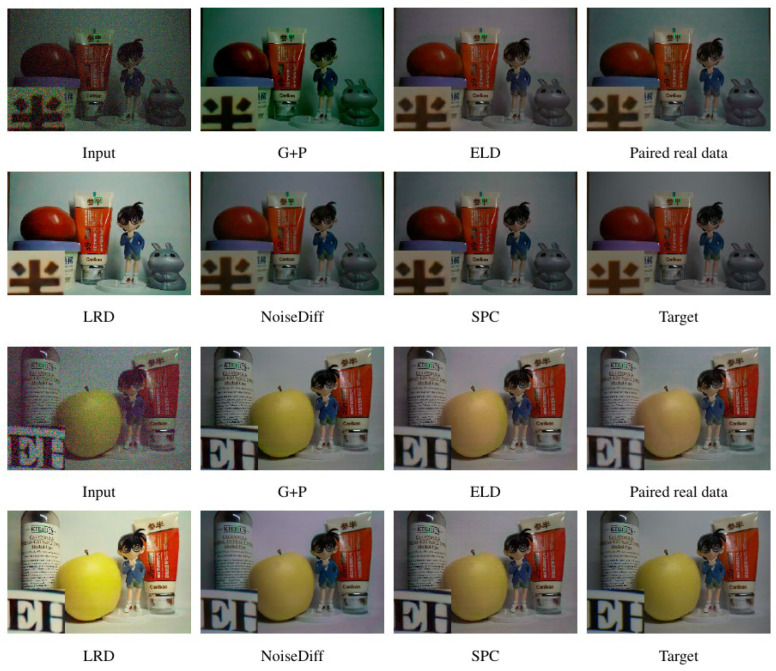
Qualitative comparison between our method and competing approaches on the LLID test set. Local regions are marked with green boxes indicate regions of interest for detailed visual comparison.

Since this study performs calibration at the single-pixel level of the camera, and given that pixel-level noise characteristics may vary even across cameras of the same model, the network trained with our method may suffer from limited cross-device robustness. Therefore, we conducted testing on the LLID captured with our own camera. Table 2 summarizes the denoising performance of different noise models on the LLID under varying exposure ratios, while Figure 5 provides corresponding visual comparisons on the same dataset. SPC denotes the results obtained by training the network with our proposed sensor pixel-calibrated (SPC) noise generation strategy, where we benchmark against the official pretrained models released by each method.

For physics-based noise models, the synthetic data generated by P-G [24] exhibits substantial discrepancies from real sensor noise, resulting in noticeable color distortions in the denoised outputs. Although ELD [8] improves the modeling of signal-independent noise, it fails to capture fixed-pattern noise and dark current shot noise, leading to suboptimal restoration performance.

For real noise-based models, their applicability is constrained by the labor-intensive process of dataset collection. Moreover, due to the inherent complexity of real-world noise [19], encounters difficulties in accurately learning the true noise distribution. Although [32] exploits the generative capability of diffusion models, its restoration results remain unsatisfactory. In contrast, our approach leverages precise physics-based noise modeling, enabling the generation of more realistic synthetic data and ultimately yielding superior image recovery quality.

#### 5.2.3. Ablation Study

To validate the effectiveness of the noise model we proposed, we conducted comparative experiments on the LLID. The image processing performance was evaluated by training the model using different noise components. Specifically, $N_{s}$ denotes photon shot noise, $N_{r}$ denotes source follower noise, $N_{H}$ denotes row noise, $N_{q}$ denotes quantization noise, $N_{FP}$ denotes fixed-pattern noise, $N_{RS}$ denotes dark current shot noise, and noD indicates that the single-pixel calibration method was not used. For example, $N_{s}+N_{r}$ means the neural network model was trained using only photon shot noise and source follower noise. We trained the network by sequentially adding different noise components and removing the single-pixel calibration method, with the resulting evaluation outcomes summarized in Table 3. The results demonstrate the effectiveness of each noise component and the single-pixel calibration method, showing that our proposed final noise model performs well.

### 5.3. PGRF Model Validation

To further improve the quality of image restoration in low-light environments, we conducted a thorough validation of the proposed PGRF and performed extensive ablation studies to verify the effectiveness of its key modules. The results demonstrate that our framework consistently achieves superior restoration performance and exhibits strong generalization capability across diverse conditions.

#### 5.3.1. Experimental Details

**Figure 6:**
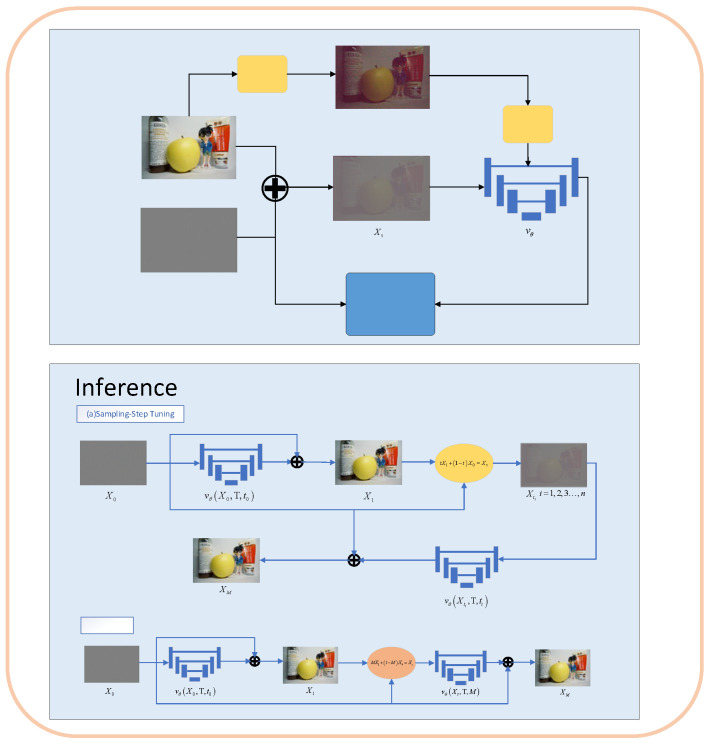
Training and testing pipeline of the proposed PGRF with the sampling search strategy.

We first used long-exposure images from the SID dataset as clean reference images for training. The SR3UNet network architecture was employed to predict the noise map $v_{\theta }$. We adopted the Adam optimizer with a batch size of 12 and an initial learning rate of $1\times 10^{-4}$, training the model for 450 k iterations. For the trained network, we applied a sampling search strategy to determine the optimal sampling time $t_{2}$. The optimally processed image was then obtained using the derived $t_{2}$. The corresponding processing pipeline is illustrated in Figure 6.

#### 5.3.2. Experimental Results

**Figure 7:**
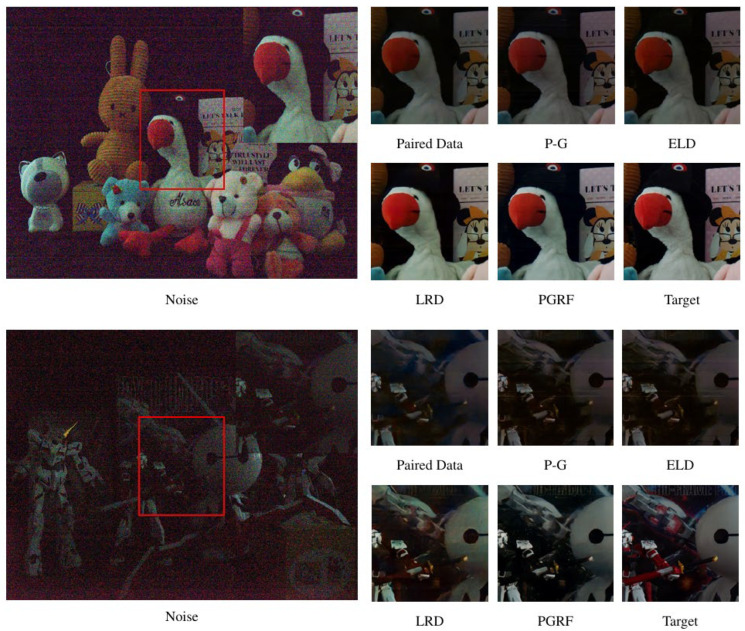
Qualitative comparison between our method and competing approaches on the ELD test set. Local regions are marked with red boxes indicate regions of interest for detailed comparison.

We further compared the denoising performance of the proposed PGRF method against existing approaches on the LLID and ELD datasets, as reported in Table 2 and Table 4. A qualitative comparison on the public ELD dataset is illustrated in Figure 7. The results of the competing methods, consistent with the analysis in Section 5.2.2, demonstrate that PGRF achieves clear superiority in image restoration. For all competing approaches, we directly employed their officially released pretrained models and implementations to obtain the results.

#### 5.3.3. Ablation Study

To further validate the effectiveness of our method, we conducted ablation experiments. In these experiments, **a** denotes the results obtained from direct training without applying the sampling search strategy, while **b** denotes the results obtained with the sampling search strategy applied. The results in Table 5 demonstrate that the optimal path search using the sampling search strategy effectively identifies better target directions, thereby improving the image processing performance of the rectified flow model.

## 6. Conclusions

This study addresses the challenge of inaccurate noise modeling in synthetic training data for low-light RAW image enhancement. From the perspective of the physical imaging mechanism of image sensors, we propose a composite noise modeling approach that integrates both additive and multiplicative noise. In particular, we extend noise calibration from the global level to the per-pixel level, thereby capturing spatially non-uniform noise characteristics induced by microscopic variations in CMOS manufacturing. This fine-grained physical noise simulation significantly improves the consistency between synthetic and real captured data in terms of noise distribution, providing higher-fidelity paired samples for training low-light image enhancement networks.

In terms of generative modeling, this work is the first to combine physical noise modeling with the rectified flow framework, resulting in the proposed PGRF (Physics-Guided Rectified Flow) for low-light image enhancement. By introducing a physics-based conditional control mechanism, the framework effectively constrains the randomness of the generation process, enabling a two-step generation from low-light images to the target enhanced images, while preserving rich detail and natural texture structures.

To validate the effectiveness of our approach, we constructed the Sony A7S II Indoor Low-Light Dataset (LLID), which includes multiple exposure settings and diverse indoor scenes. Experimental results demonstrate that PGRF achieves substantial improvements in both visual quality and inference efficiency compared with existing methods for low-light RAW image enhancement.

Overall, this study overcomes the limitations of conventional noise modeling and introduces a novel paradigm that integrates physics-based priors with efficient generative models, demonstrating academic value and practical potential.

## Figures and Tables

**Table 1:** Comparison of results obtained using two fitting methods on the PGRF dataset.

Model	Index	100	200
y=at	PSNR/SSIM	41.221/0.958	38.510/0.935
$y=a\sqrt{t}$	PSNR/SSIM	41.811/0.968	39.498/0.953

**Table 2:** Denoising results for RAW images on the LLID. The best results are highlighted in red, and the second-best results are highlighted in blue.

		Physics-Based				Real Noise-Based		
Ratio	Index	PGRF	SPC	ELD [8]	P-G [24]	LRD [19]	NoiseDiff [32]	Paired Data [3]
100	PSNR	42.962	4 1.811	40.364	40.610	40.565	41.272	40.245
	SSIM	0.975	0.968	0.964	0.941	0.967	0.970	0.963
200	PSNR	40.805	39.498	38.650	37.268	39.036	39.010	38.505
	SSIM	0.963	0.953	0.950	0.878	0.9 56	0.954	0.950

**Table 3:** Comparison of ablation study results on the proposed method. The best results are highlighted in red.

Model	Index	100	200
$N_{s}+N_{r}$	PSNR/SSIM	40.635/0.937	37.233/0.872
$N_{s}+N_{r}+N_{FP}$	PSNR/SSIM	40.884/0.965	38.464/0.947
$N_{s}+N_{r}+N_{FP}+N_{H}$	PSNR/SSIM	40.934/0.966	38.476/0.948
$N_{s}+N_{r}+N_{FP}+N_{H}+N_{q}$	PSNR/SSIM	41.149/0.966	38.574/0.947
$N_{s}+N_{r}+N_{FP}+N_{H}+N_{q}+noD$	PSNR/SSIM	40.805/0.963	38.438/0.945
$N_{s}+N_{r}+N_{FP}+N_{H}+N_{q}+N_{RS}$	PSNR/SSIM	41.81 1 /0.9 68	39. 498 /0.9 53

**Table 4:** Denoising results for RAW images on the ELD dataset. The best results are highlighted in red, and the second-best results are highlighted in blue.

		Physics-Based			Real Noise-Based		
Ratio	Index	PGRF	ELD [8]	P-G [24]	LRD [19]	Noisediff [32]	Paired Data [3]
100	PSNR	45 .945	44.705	43.106	44.912	45.206	44.014
	SSIM	0.972	0.967	0.922	0.975	0.968	0.968
200	PSNR	43.920	42.883	39.876	42.752	42.636	42.101
	SSIM	0.95 4	0.951	0.855	0.959	0.942	0.947

**Table 5:** Comparison of ablation study results evaluating the effect of the sampling search strategy.

Model	Index	a	b
100	PSNR/SSIM	45.858/0.970	45.945/0.972
200	PSNR/SSIM	43.902/0.954	43.920/0.954

## Data Availability

The raw data supporting the conclusions of this article will be made available by the authors on request.
